# Iron‐doped nanozymes with spontaneous peroxidase‐mimic activity as a promising antibacterial therapy for bacterial keratitis

**DOI:** 10.1002/SMMD.20240004

**Published:** 2024-05-22

**Authors:** Xiwen Geng, Nan Zhang, Zhanrong Li, Mengyang Zhao, Hongbo Zhang, Jingguo Li

**Affiliations:** ^1^ Henan Provincial People's Hospital People's Hospital of Zhengzhou University Zhengzhou China; ^2^ Pharmaceutical Sciences Laboratory Faculty of Science and Engineering Åbo Akademi University Turku Finland; ^3^ Turku Bioscience Centre University of Turku and Åbo Akademi University Turku Finland; ^4^ Henan Eye Hospital Zhengzhou China

**Keywords:** antibacterial, antimicrobial‐resistant, bacterial keratitis, nanozyme, ROS

## Abstract

The development of non‐antibiotic pharmaceuticals with biocompatible and efficient antibacterial properties is of great significance for the treatment of bacterial keratitis. In this study, we have developed antibacterial iron‐doped nanozymes (Fe^3+^‐doped nanozymes, FNEs) with distinguished capacity to fight against bacterial infections. The iron‐doped nanozymes are composed of Fe^3+^ doped zeolitic imidazolate framework‐8 (Fe/ZIF‐8) and polyethylene imide (PEI), which were functionally coated on the surface of Fe/ZIF‐8 and imparted the FNEs with improved water dispersibility and biocompatibility. FNEs possess a significant spontaneous peroxidase‐mimic activity without the need for external stimulation, thus elevating cellular reactive oxygen species level by catalyzing local H_2_O_2_ at the infection site and resulting in bacteria damaged to death. FNEs eliminated 100% of *Staphylococcus aureus* within 6 h, and significantly relieved inflammation and bacterial infection levels in mice bacterial keratitis, exhibiting higher bioavailability and a superior therapeutic effect compared to conventional antibiotic eye drops. In addition, the FNEs would not generate drug resistance, suggesting that FNEs have great potential in overcoming infectious diseases caused by antimicrobial resistant bacteria.

## Graphical abstract

A functionalized antibacterial iron‐doped nanozyme with spontaneous peroxidase ‐mimic activity was fabricated to treat bacterial keratitis. The iron‐doped nanozymes could elevate cellular reactive oxygen species level and result in bacteria damaged to death, thus exhibiting an excellent therapeutic effect in bacterial keratitis.

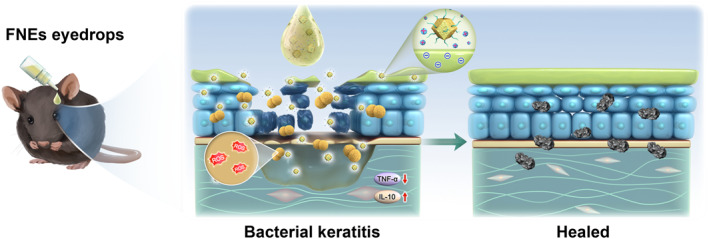

**Key points**

- Iron‐doped nanozymes (FNEs) were fabricated with antibacterial and anti‐inflammatory ability for effective treatment of bacterial keratitis.
- FNEs could mimic peroxidase activity and spontaneously catalyze H_2_O_2_ to produce much more reactive oxygen species at the infection site to eliminate bacteria without external stimulation.
- The FNEs exhibited distinguished biocompatibility with ocular cells and tissue, devoid of drug resistance issues, and possessed superior therapeutic potential in comparison to conventional antibiotics.

## 1 INTRODUCTION

Bacterial keratitis, which may cause corneal perforation and even severe vision impairment, is a common corneal inflammation induced by bacterial infections.^1^, ^2^, ^3^ The most widely used therapy for bacterial keratitis is antibiotic‐based eye drops.^4^ However, owing to the abuse of antibiotics, antimicrobial resistant (AMR) bacterial infections are rapidly emerging worldwide and overshadowing the prospects of antibiotic treatments.^3^, ^5^, ^6^, ^7^, ^8^ Therefore, the exploration of novel strategies that function through other mechanisms to solve AMR infections becomes a tremendous challenge. To address this challenge, substantial efforts are devoted to novel nano‐therapies including laser, ultrasonic, or X‐ray, which have been reported to achieve high antibacterial efficiency.^9^, ^10^ Nevertheless, these approaches are likely to cause irritation or impairment to human eyes.^11^, ^12^, ^13^ Herein, antibacterial nanozyme catalyzing reactive oxygen species (ROS) is considered as a preferred alternative bactericidal therapy in combatting bacterial infection because of their benign biocompatibility and low likelihood of inducing AMR.^14^, ^15^, ^16^, ^17^, ^18^

With the rapid development of materials science, nano‐chemistry and nano‐biotics, the application of nanozyme‐based drugs in the biomedical field has become a new research hotspot.^19^ Nanozymes are nanomaterials with enzyme‐like activity that efficiently catalyze substrate chemical reactions under physiological conditions and follow similar enzyme kinetics and mechanisms as native enzymes.^20^, ^21^, ^22^, ^23^ Profiting from the merits of nanomaterials, nanozymes can overcome natural enzymes' shortcomings such as intricate production routines, high cost and low permeability, leading to widespread applications in areas such as biomedical sensing, therapeutics, and environmental remediation, especially offering great possibilities for the development of novel antimicrobial drugs.^24^, ^25^ Therefore, it is of great clinical significance for the treatment of bacterial keratitis to construct new nano‐enzyme‐based drugs to kill bacteria and innovate the research and development strategy of antibacterial drugs efficiently and collaboratively.^26^ Metal‐organic frameworks (MOFs) are a kind of promising biomedical material with unique properties of large specific surface area, flexible functional groups, ultrahigh porosity, and variable porous structures.^27^, ^28^ The size‐controllable pores of MOFs can not only offer a hydrophobic confined environment but also provide an ordered arrangement of active catalytic sites that allow great accessibility to high‐density substrates.^29^ Hence, MOF materials are regarded as potential candidates for enzyme mimics. There are some articles reporting the applications of nanozymes constructed with MOF materials. For instance, Liu et al. have synthesized a Zn‐based nanozyme using a zeolitic‐imidazolate framework (ZIF‐8), which has effective peroxidase (POD) ‐like activity and enhanced the efficiency of ROS generation, consequently achieving effective treatment of bacteria wound infection.^30^ Fe‐based nanomaterials are widely used in biomedical applications due to their paramagnetism. Gao et al. first fabricated the magnetite (Fe_3_O_4_) nanoparticles with intrinsic POD‐like activity due to the rich Fe^3+^ and Fe^2+^ on the surface, and soon Fe‐based nanozymes with the POD‐like activity were fabricated since then.^31^ Notably, plenty of Fe‐based nanoparticles have been approved by the United States Food and Drug Administration for clinical translation. Accordingly, Fe‐doped MOFs can not only have enhanced ROS generation function but also possess outstanding biocompatibility.^32^ Nevertheless, to the best of our knowledge, Fe‐doped MOFs have seldom been reported for use in ocular tissue disinfection research, and the insolubility of MOFs greatly impedes their further development in the biomedical applications.^33^, ^34^ Therefore, we have been exploring the antimicrobial properties of Fe‐doped MOFs for applications in ocular infection therapies.

**FIGURE 1:**
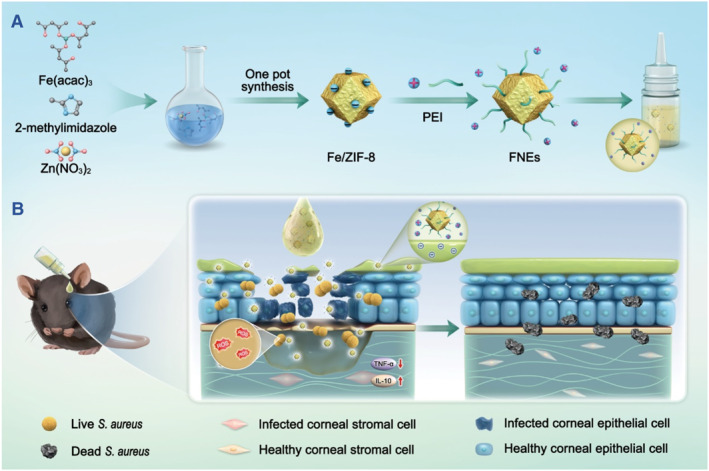
Schematic diagram of FNEs fabrication and application in bacterial keratitis.

Herein, we have developed the iron‐doped nanozymes (Fe^3+^‐doped nanozymes, FNEs) with excellent ROS generation capacity to fight against bacterial infection. The FNEs are composed of Fe^3+^ doped ZIF‐8 (Fe/ZIF‐8) and polyethylene imide (PEI), which was functionally coated on the surface of Fe/ZIF‐8 and imparted the FNEs with improved water dispersibility and biocompatibility. FNEs possess a significant spontaneous POD‐like activity without the need for external stimulation, thus elevating cellular ROS level by catalyzing local H_2_O_2_ at the infection site (Figure 1). In this study, the bactericidal effect and possible mechanisms of FNEs were investigated and a mice bacterial keratitis model was employed to evaluate the therapeutic efficacy. Furthermore, the biosafety and anti‐resistance potentials were also discussed.

## 2 METHODS AND MATERIALS

### 2.1 Materials

2‐methylimidazole, zinc nitrate hexahydrate (Zn(NO_3_)_2_·6H_2_O), Fe(acac)_3_, polyethylene imide (PEI, 1800 Da) were purchased from Shanghai Aladdin Biochemical Technology Co., Ltd (Shanghai, China).

### 2.2 Synthesis of FNEs nanozyme

1.68 g Zn(NO_3_)_2_ and 0.7 g Fe(acac)_3_ were dissolved in 80 mL methanol. 3.70 g 2‐methylimidazole dissolved in 80 mL methanol was poured into the above solution, and stirred quickly overnight. After that, the precipitation was collected by centrifuge and washed with methanol three times. Fe^3+^ doped ZIF‐8 (Fe/ZIF‐8) powder was obtained after vacuum drying at 60°C. FNEs were obtained through the PEI coating process. Fe/ZIF‐8 was dissolved in pure water, PEI was added and stirred overnight; when the solution became transparent without precipitation, the PEI modified Fe/ZIF‐8 was obtained. The corresponding product was denoted as FNEs.

### 2.3 Characterization of Fe/ZIF‐8 and FNEs nanozyme

The microscopic morphology of the Fe/ZIF‐8 was captured by a field emission scanning electron microscope (SEM, ZEISS) and a TalosF200S transmission electron microscope (TEM, FEI). X‐ray diffraction analysis was carried out by an X‐ray powder diffractometer (XRD, Empyrean). The Malvern particle size potentiometer measured the *ζ* potential of Fe/ZIF‐8 and FNEs. X‐ray photoelectron spectroscopy (XPS) spectra were obtained using an X‐ray photoelectron spectrometer (AXIS Supra, Shimadzu/Kratos) with Al Kα radiation as an X‐ray source.

The POD‐like activity of FNEs. TMB with pH of 5.5 was used to analyze the production of •OH, and the solution containing FNEs (180 μL, final concentration: 0, 25, 50, 100, 200, 500, 1000 mg/mL), TMB (40 mM in DMSO, 10 μL), and H_2_O_2_ (200 mM, 10 μL) was mixed. The colors of the solutions were imaged and the final absorbance at 652 nm was measured by the microplate reader. The Ti(SO_4_)_2_ method was used to evaluate the H_2_O_2_ catalytic activity of FNEs. FNEs (1000 μg/mL, 100 μL) was mixed with H_2_O_2_ (20 mM, 100 μL). The reaction was terminated with Ti(SO_4_)_2_ (400 μL), and the absorption at 420 nm can be used to quantify H_2_O_2_.

### 2.4 In vitro antibacterial assays

#### 2.4.1 Bacterial strains, media, growth conditions

*Staphylococcus aureus* (*S. aureus*) used in this study was supplied by the Henan Eye Institute. Bacteria were cultivated on trypticase soy agar (TSA) plates at 37°C. The liquid culture medium for *Staphylococcus aureus* was trypticase soy broth (TSB), and the bacteria cells were incubated in a shaker at 37°C. TSA, TSB medium and tobramycin were from Sigma‐Aldrich (St. Louis, MO, USA).

#### 2.4.2 Determination of minimum inhibitory concentration

The measurement of MICs of FNEs and TOB was performed for the evaluation of the inhibitory ability on bacterial growth. The bacteria were seeded with TSB medium onto a 96‐well plate with a preliminary optical density at 600 nm wavelength (OD_600_) of 0.1. FNEs (TOB) were added to the plate and obtained final concentrations of 0–100 μg/mL with or without H_2_O_2_ (10 μM). After 12 h of incubation at 37°C, the OD_600_ values were recorded by the Cytation5 Microplate Reader (Biotek Winooski).

#### 2.4.3 Live/dead fluorescence staining

For the determination of bacterial morphology and viability, bacterial cells were collected, washed and resuspended in fresh TSB medium with or without FNEs (31.5 μg/mL) and H_2_O_2_ (10 μM), respectively. After incubation for 4 h at 37°C, the bacteria cells were collected by centrifugation and stained with SYTO and propidium iodide (PI) from the Live/Dead BacLight Bacterial Viability kits (Invitrogen, USA) for 15 min and washed twice with phosphate‐buffered saline (PBS). Then, the bacteria cells were visualized by confocal microscopy (Leica TCS SP5).

### 2.5 Measurement of reactive oxygen species

The burst generation of ROS was measured using 2,7‐Dichlorodi‐hydrofluorescein diacetate (DCFH‐DA, Sigma‐Aldrich). 1 mL bacteria cells in the logarithmic phase were collected by centrifugation with 5000 rpm for 5 min and washed twice with PBS. The precipitate was suspended with 0.5 mL of DCFH‐DA (10 μM) and incubated at 37°C for 30 min in dark conditions. Then, the bacteria cells were recollected by centrifugation and resuspended in 1 mL PBS. Aliquots (200 μL) were transferred to a 96‐well plate. The excitation and emission were measured at 490 and 530 nm, respectively, by Cytation5 Microplate Reader.

### 2.6 In vivo antibacterial activity studies

Adult C57BL/6 male mice (6–8 weeks, 17–22 g) were used in animal studies. The mice were first anesthetized with 4% chloral hydrate (10 mg/g), and 0.4% oxybuprocaine hydrochloride was used for topical anesthetization around ocular tissues. Then, 2 mm‐diameter scratches were constructed on the center corneas of the right eye of the mice and reached the stroma layer using an optimum knife (straight 45‐degree, Beaver, China). The left eye was left untreated as a control. Subsequently, the aliquots (5 μL, 10^8^ CFU/mL) *S. aureus* cells were spread to the scratched area to establish the mice bacterial keratitis model. The next day, the modeled mice were randomly divided into 3 groups applied with saline, TOB (5 μL, 50 μg/mL) and FNEs (5 μL, 50 μg/mL) eye drop solutions, respectively. TOB was used as a positive control. Eye drops were applied to the left eye of the mice 3 times daily for 7 days. Corneal pathological observations were photographed and recorded every other day for further analysis. After day 7, the mice were sacrificed, and the eyeball tissues were collected. Part of the eyeballs was fixed in 4% formaldehyde for further analysis, and others were cultured onto the TSB plate for colony counting. Additionally, Hematoxylin‐eosin staining (H&E staining) was performed to evaluate epithelization, and Gram staining for the bactericidal effect. Immunohistochemistry staining by primary antibodies against interleukin 10 (IL‐10) and tumor necrosis factor *α* (TNF‐*α*) were utilized for inflammation evaluation.

### 2.7 Pharmacokinetic and statistical analyses

Data in this study were analyzed using GraphPad Prism software (GraphPad Software Inc.), and presented as the means ± standard errors. *p* values ≤ 0.05 were considered statistically significant.

## 3 RESULTS AND DISCUSSION

### 3.1 Characterization of Fe/ZIF‐8 and FNEs

**FIGURE 2:**
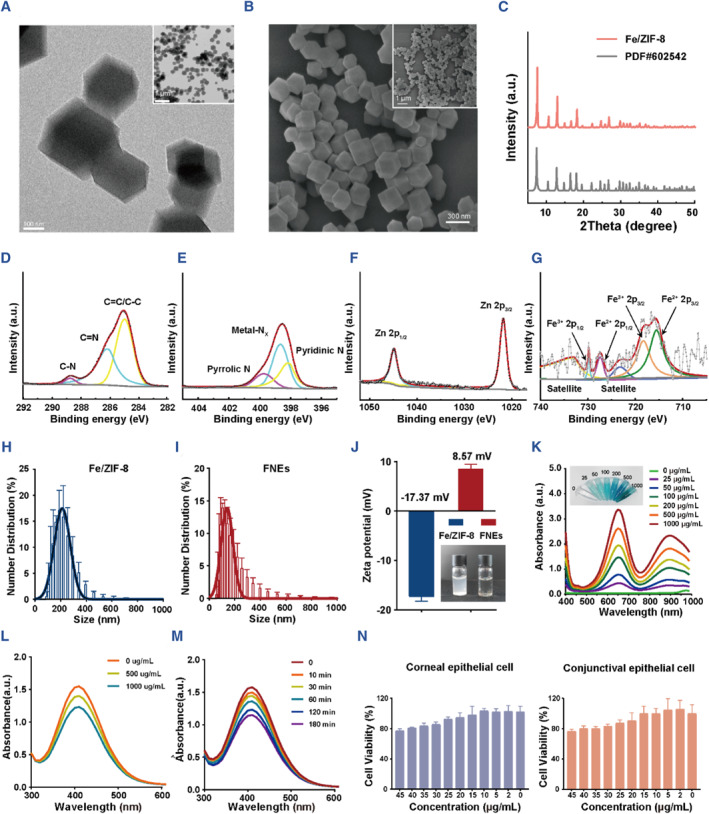
Characterization of Fe/ZIF‐8 and FNEs. (A) TEM images and (B) SEM images of Fe/ZIF‐8. (C) XRD pattern of Fe/ZIF‐8. C (D), N (E), Zn (F) and Fe (G) XPS spectrums of Fe/ZIF‐8. Particle size of Fe/ZIF‐8 (H) and FNEs (I). (J) Zeta potential of Fe/ZIF‐8 and FNEs. (K) Absorbance curves of TMB‐oxide after incubation with FNEs of varied concentrations. The image inserted is the corresponding TMB‐oxide color change. FNEs concentration‐dependent (L) and time‐dependent (M) decomposition of H_2_O_2_ using Ti(SO_4_)_2_. (N) Human corneal epithelial cell and human conjunctival epithelial cell viabilities after culturing with FNEs for 24 h.

Generally, Fe/ZIF‐8 was fabricated using a simple ‘one pot’ method.^35^ With Zn^2+^ as the metal center and 2‐methylimidazole as the organic ligand, periodic zeolite imidazole‐like skeleton material (ZIF‐8) was self‐assembled in methanol solution. Zn^2+^ and four deprotonated 2‐methylimidazole were bridged with N atoms at position 1 and three of the imidazole ring to form a six‐membered ring cage, and a topological structure was formed by a single four‐membered ring. The Fe^3+^ are encapsulated in ZIF‐8 pores. The TEM and SEM images of Fe/ZIF‐8 exhibited a typical regular dodecahedron structure with uniform size (Figure 2A,B). XRD analysis of Fe/ZIF‐8 was further conducted; the result showed that Fe/ZIF‐8 had similar XRD patterns with the standard crystal structure of ZIF‐8 (Figure 2C), verifying that Fe/ZIF‐8 was successfully synthesized. Size engineering of MOF materials has a significant effect on the antibacterial performance, and smaller size may make it easier to bind to substrates and penetrate the bacterial membranes.^36^, ^37^ The Malvern particle size potentiometer result showed that the size of Fe/ZIF‐8 was around 200 nm that was consistent with the TEM and SEM images (Figure 2H). The X‐ray photoelectron spectroscopy (XPS) result confirmed that Fe/ZIF‐8 had the components of C, N, Zn and Fe. The high‐resolution image of C 1s in Figure 2D can be deconvoluted into three peaks at 285.0, 286.1 and 288.8 eV, matching with C=C/C‐C, C=N and C‐N, respectively.^38^ The N 1s spectrum in Figure 2E can be attributed to three main contributions: pyridinic N at 398.1 eV, Metal‐N at 398.7 eV and pyrrolic N at 399.7 eV.^39^ The presence of the Metal‐N peak further indicated that Fe and Zn coordinated with N species, revealing the successful synthesis of Fe/ZIF‐8.^40^, ^41^ The XPS spectrum of Zn *2p* can be fitted to two curves with the peaks located at 1021.8 and 1044.8 eV (Figure 2F).^42^ As shown in Figure 2G, the XPS spectrum of Fe *2p* also demonstrated the existence of Fe^2+^ and Fe^3+^. The above characterizations indicated that Fe^3+^‐doped ZIF‐8 structure was successfully synthesized.

Since Fe/ZIF‐8 is negatively charged and dispersed instead of dissolved in water, we further modified the product with PEI to form iron‐doped nanozymes (Fe^3+^‐doped nanozymes, denoted as FNEs). FNEs have a similar size to Fe/ZIF‐8 and PEI modification made FNEs positively charged (+8.57 mV) and more conducive to enhancing the affinity to the negatively charged cell membrane; in addition, PEI modification also effectively remedied the insolubility in water of Fe/ZIF‐8 (Figure 2I,J).

H_2_O_2_ is the most common reactive oxygen molecule in living systems, and is also the donor of ROS, catalyzed by CAT and POD.^43^ H_2_O_2_ can react with Ti(SO_4_)_2_ to produce yellow peroxide‐Ti complex precipitation, which has a characteristic peak at 415 nm, thus the concentration of H_2_O_2_ can be quantitatively detected by the change of absorbance value.^44^ We used Ti(SO_4_)_2_ to determine the concentration of H_2_O_2_ to explore the catalase POD‐like activity (Fenton reaction) of FNEs. As depicted in the Figure 2L,M, with the increase in the FNEs concentration and time, the absorbance at 415 nm decreased continuously, indicating that FNEs can effectively catalyze the Fenton reaction to produce •OH. Meanwhile, the chromogenic reaction of •OH and 3,3′,5,5′‐tetramethylbenzidine (TMB) was performed to further characterize the POD‐mimic performance of the FNEs. The color and absorbance variations of TMB‐oxide demonstrated that the oxidation of TMB can be catalyzed by FNEs in a concentration‐dependent way as a POD‐like nanozyme (Figure 2K). All the data confirmed the ability of FNEs to produce •OH by catalyzing H_2_O_2_.

Since the biocompatibility of pharmaceuticals is critical for biomedical applications, we examined the biocompatible effects of FNEs on the viability of human corneal and conjunctiva epithelial cells. As shown in Figure 2N, the survival rate of human corneal and conjunctival epithelial cells remained over 80% after incubation with FNEs for 24 h at a concentration of 40 μg/mL, indicating that FNEs had satisfied biocompatibility. We also tested ROS levels in the corneal epithelial cells using DCFH‐DA to clarify the damage difference between corneal cells and bacteria (Figure S1A). As shown in Figure S1B, after being treated with FNEs, the ROS level in cells did not change. However, after treatment with FNEs, the ROS level in bacteria doubled compared to the untreated bacteria (Figure S1C), proving that the process of ROS generation does not cause damage to the cornea.

### 3.2 Antibacterial properties in vitro

**FIGURE 3:**
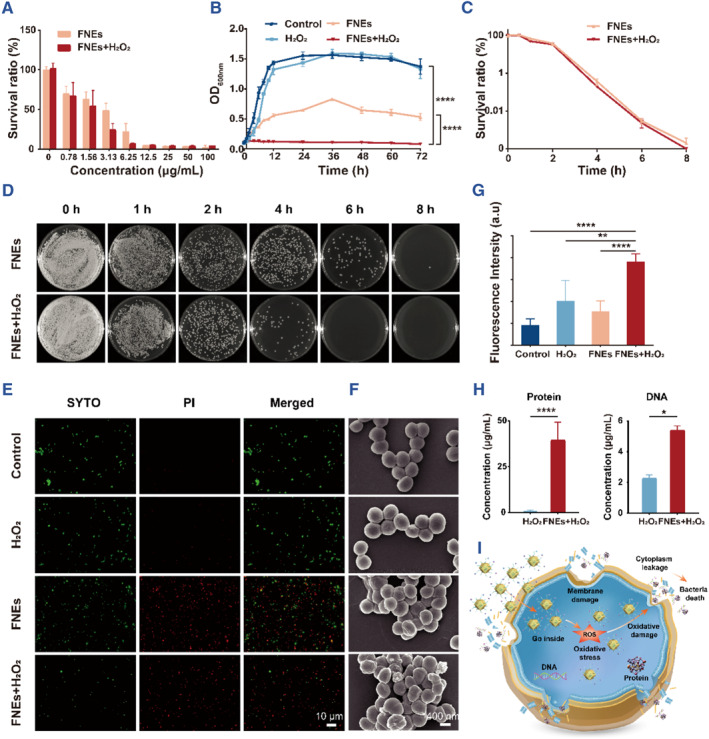
Antibacterial effect of FNEs in vitro. (A) MICs of FNEs with or without H_2_O_2_ (10 nM) of *S. aureus*, (B) Growth kinetics of *S. aureus* with different treatments, (C) the corresponding growth‐inhibit curve, and (D) the corresponding plate counting assays. (E) Fluorescent Live/Dead images of *S. aureus* and (F) the corresponding SEM images with different treatments. (G) ROS generation level of different treatments in *S. aureus*. (H) Protein leakage and DNA leakage of *S. aureus* with different treatments. (I) Antibacterial mechanism schematic diagram of FNEs against *S. aureus*. The asterisks indicate significant differences (*t*‐test, *p ≤ 0.05; **p ≤ 0.01; ****p ≤ 0.0001).

After the synthesis and characterization, the antibacterial properties of FNEs were further studied using *S. aureus*. Tobramycin (TOB), which is the clinical antibacterial first‐line drug for conjunctivitis, keratitis and other bacterial eye infections, served as the positive control in this study.^45^ Thus, we examined the minimum inhibitory concentrations (MICs) of both FNEs and TOB. It can be seen that MIC of FNEs + H_2_O_2_ (6.25 μg/mL) was only half that of FNEs (12.5 μg/mL), and both of them were much lower than the MIC of TOB (25 μg/mL) (Figure 3A, Figure S2A). The results showed that FNEs had a markedly better antibacterial activity than TOB and the existence of H_2_O_2_ increased FNEs' antibacterial efficiency.

We further performed the growth‐kinetic assays; bacteria cells were incubated with saline (control), H_2_O_2_, FNEs, FNEs + H_2_O_2_ and TOB for 72 h, separately. As shown in the Figure 3B, saline and H_2_O_2_ had no effect on bacteria growth, the growth of cells treated with FNEs was limited and did not reach the logarithmic phase, while bacteria cells treated with FNEs + H_2_O_2_ did not grow at all. Surprisingly, cell growth in the TOB group was limited initially and then recovered to normal after 24 h (Figure S2B), indicating that bacteria cells have the resistance potential to TOB, which also explains why clinical first‐line antibiotics are not working always well. The growth‐inhibit assays showed that the bacteria cells exhibited a considerably sharper decrease after treatment with 5 × MIC of FNEs (with or without H_2_O_2_) for 2 h, and after 8 h no living cells can be observed, but those treated with 5 × MIC of TOB still cannot be completely eliminated. This phenomenon implied that FNEs had better antibacterial properties than TOB (Figure 3C,D, Figure S2C,D).

As shown in Figure 3E, the bacteria were stained with SYTO and propyl iodide (PI) to further assess the antibacterial effect.^46^ SYTO (green fluorescent) is a membrane‐permeable dye that can label all bacteria, while PI (red fluorescent) is a membrane‐impermeable dye that only labels bacteria with membrane damage. In this study, bacteria cells were incubated with saline (control), H_2_O_2_, FNEs and FNEs + H_2_O_2_ for 4 h. No significant difference in bacteria viability was observed between the control and H_2_O_2_ groups, and the survival of *S. aureus* in FNEs and FNEs + H_2_O_2_ was significantly reduced. The morphological changes of *S. aureus* after treatments were determined by SEM. The results showed that both FNEs + H_2_O_2_ and FNEs could cause obvious collapse of the bacterial surface, which was the main cause of bacterial death. FNEs + H_2_O_2_ group exhibited the most severe morphological damage, indicating the superior bactericidal ability (Figure 3F). After that, we measured the protein leakage as well as nucleic acid leakage (Figure 3H) of *S. aureus* cells after different treatments to further investigate the damage of FNEs to the bacterial wall and membrane. The result showed that compared with the control group, the protein and nucleic acid leakage in the FNE group was significantly increased, which indicated that FNEs could damage the integrity of the bacteria.

Since the FNEs has such remarkable antibacterial properties and H_2_O_2_ promoted the performances of FNEs, we assumed that the enzyme property plays the essential role and further investigated its POD‐mimic activity with *S. aureus*. DCFH‐DA (2, 7‐dichlorodi‐hydrofluorescein diacetate) is the most sensitive and commonly used probe for the detection of ROS, which does not fluoresce itself. When entering cells, DCFH‐DA is hydrolyzed to DCFH (2, 7‐dichlorodihydrofluorescein), and can be oxidized to strong green fluorescence of DCF model (2, 7‐dichlorofluorescein) after reacting with ROS in cell.^47^ As shown in the Figure 3G, the presence of FNEs and H_2_O_2_ can produce much more ROS than other groups, indicating that FNEs can act as a POD‐mimic nanozyme to catalyze the oxidation of DCF, confirming the POD‐mimic activity of FNEs to catalyze H_2_O_2_ to ROS production.

Overall, the antibacterial mechanism of FNEs mainly relies on the disruption of bacterial integrity, as well as the induction of intracellular oxidative stress (Figure 3I). Firstly, positively charged FNEs electrostatically interacted with the negatively charged surface of the bacteria, subsequently changed the permeability, and resulted in the destruction of the cell membrane and wall ultimately.^48^ Secondly, the entry of FNEs into bacteria stimulates the enhanced conversion of H_2_O_2_ to ROS, especially •OH, and induces systematically oxidative cellular damage, primarily including lipid peroxidation, protein carbonylation and DNA oxidations, all of which are highly destructive to the biological systems and jeopardizes cellular integrity of bacteria and ultimately lead to cell death.^43^

### 3.3 Antibacterial therapeutic effects in vivo

**FIGURE 4:**
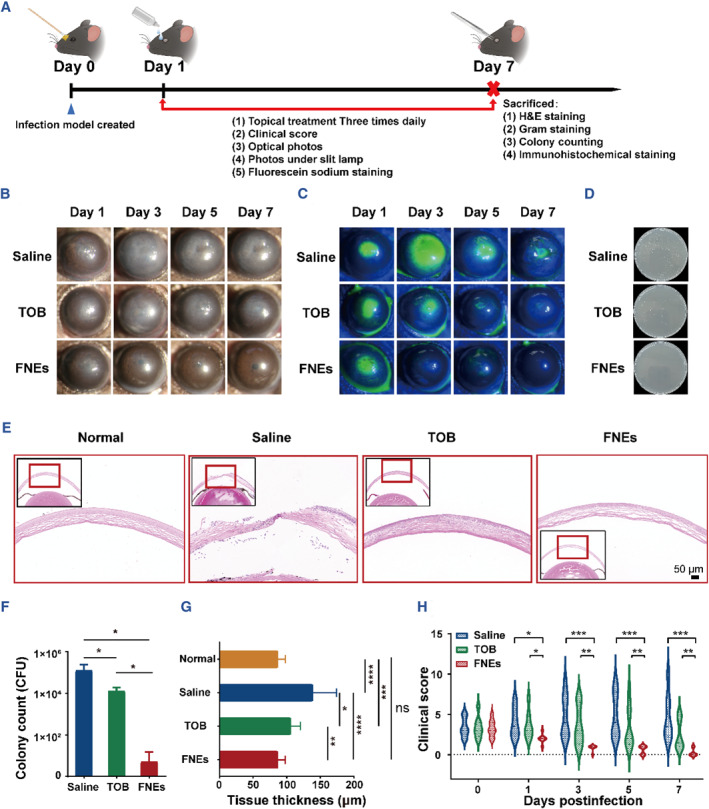
In vivo antibacterial evaluation of FNEs. (A) Schematic diagram of bacterial keratitis model establishment and drug administration. Representative photos (B) and fluorescein stained photos under slit‐lamp micrographs (C) of corneas with different treatments. (D) Plate photos of bacteria recultivated from the infected cornea after different treatments, and (F) the corresponding histogram of the bacterial number. Representative H&E staining images (E) and the corresponding corneal thickness (G) of the infected cornea after different treatments for 7 days. (H) Histogram of clinical scores for keratitis after different treatments. The asterisks indicate significant differences (*t*‐test, *p ≤ 0.05; **p ≤ 0.01; ***p ≤ 0.001).

Due to the superior antibacterial properties and great biocompatibility in vitro, the FNEs were further applied to a mice bacterial keratitis model. Detailed steps are shown in Figure 4A. As shown in Figure 4B,C and Figure S3, corneal damage, edema, and apparent haze were observed in all groups on day 1. On day 3, corneas in the FNE group showed reduced haze and slight cornea recovery, and no further corneal damage and increased haze were observed in the TOB group. However, the control group showed more severe corneal edema and haze, and the fluorescein sodium staining photos showed that the corneal damage expanded. On day 7, corneas of the FNEs group showed clear and intact corneal structure, the TOB group showed a substantial reduction in haze, and the control group remained cloudy. Compared to the control group, eyes treated with TOB showed lower clinical scores, and the FNEs group showed the lowest score of 0, indicating that the eyes recovered from the disease (Figure 4H).

**FIGURE 5:**
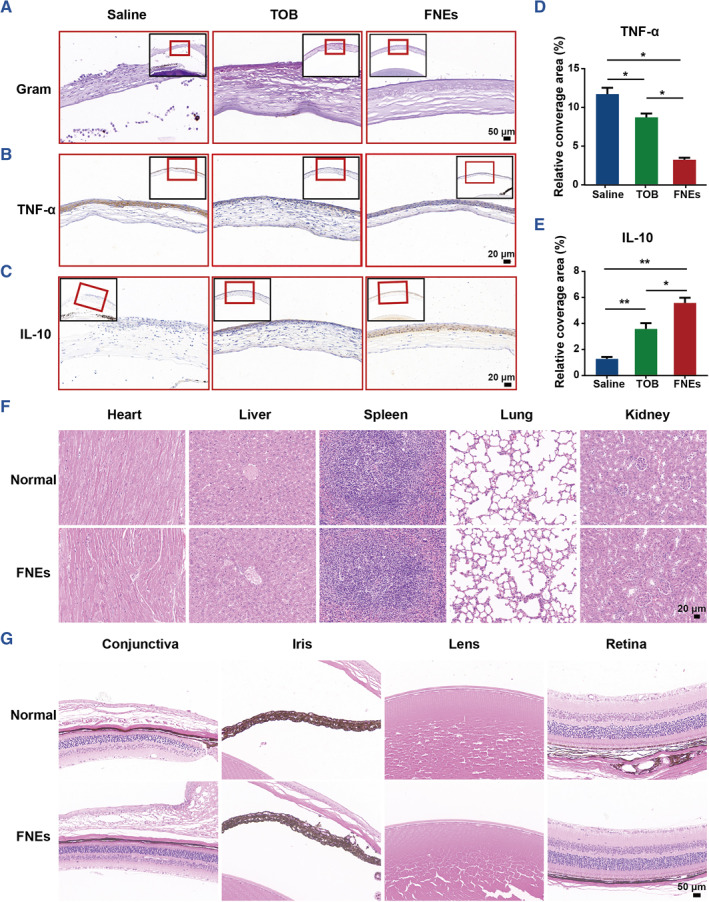
In vivo immunological evaluation of FNEs. (A) Representative Gram staining images of the infected cornea after different treatments. (B) Representative TNF‐*α* immunohistochemistry images of the infected cornea after different treatments and (D) the corresponding histogram. (C) Representative IL‐10 immunohistochemistry images of the infected cornea after different treatments and (E) the corresponding histogram. (F) Representative H&E staining images of heart, liver, spleen, lung and kidney of mice after treatment with or without FNEs. (G) Representative H&E staining images of conjunctiva, iris, lens and retina after treatment with or without FNEs. The asterisks indicate significant differences (*t*‐test; *p ≤ 0.05; **p ≤ 0.01).

In addition, in order to determine the antibacterial efficiency of each group, Gram staining and tissue recultivation were performed on the eye tissue of each group, and it was found that the number of bacteria in FNEs group was almost negligible on day 7; the number of bacteria in TOB group was also significantly reduced compared with the control group (Figures 4D,F and 5A).

The infected corneal tissues were collected on day 7 and Hematoxylin‐eosin staining (H&E staining) was carried out to assess the tissue morphology. As shown in Figure S3, corneal tissues of the control group still showed significant corneal edema in slit‐lamp micrographs on day 7; correspondingly, H&E images showed that a large number of infiltrated inflammatory cells and a significant increment in corneal thickness were observed in the control group, a small number of inflammatory cells in the TOB group, while none in the FNEs group. In addition, the corneal thickness and structure of the FNEs group had no difference with that of normal corneal tissue (Figure 4E,G), indicating that corneas of FNEs group had already recovered on day 7.

To demonstrate the antiinfection performance of the FNEs, immunohistochemical analysis was performed to investigate the expressions of TNF‐α and IL‐10. TNF‐α is a typical pro‐inflammatory factor, the expression level of which can reflect the inflammation level caused by bacteria infection.^49^ IL‐10 is essential for the integrity and maintenance of tissue epithelium, suppressing pro‐inflammatory responses and limiting tissue destruction caused by inflammatory response caused by infections.^50^ As shown in Figure 5B–E, TNF‐*α* expression level was significantly lower in the FNEs group, while the IL‐10 level was the highest among the three groups. This might be attributed to the outstanding antibacterial effect of FNEs, which effectively protected the corneas against further bacterial infections and avoided severe inflammatory responses. Meanwhile, we evaluated the in vivo biotoxicity of FNEs. Tissues of five major organs and major eye associated structures of mice with or without FNEs treatment were collected and stained with H&E.^51^ All tissues showed integrated and normal morphology, and no significant differences were observed (Figure 5F,G), suggesting that FNEs are nontoxic and have satisfied locally and systemically biocompatibility. The aforementioned results demonstrated that the presented FNEs are a promising therapy for bacterial keratitis.

### 3.4 Irritation test in vivo

**FIGURE 6:**
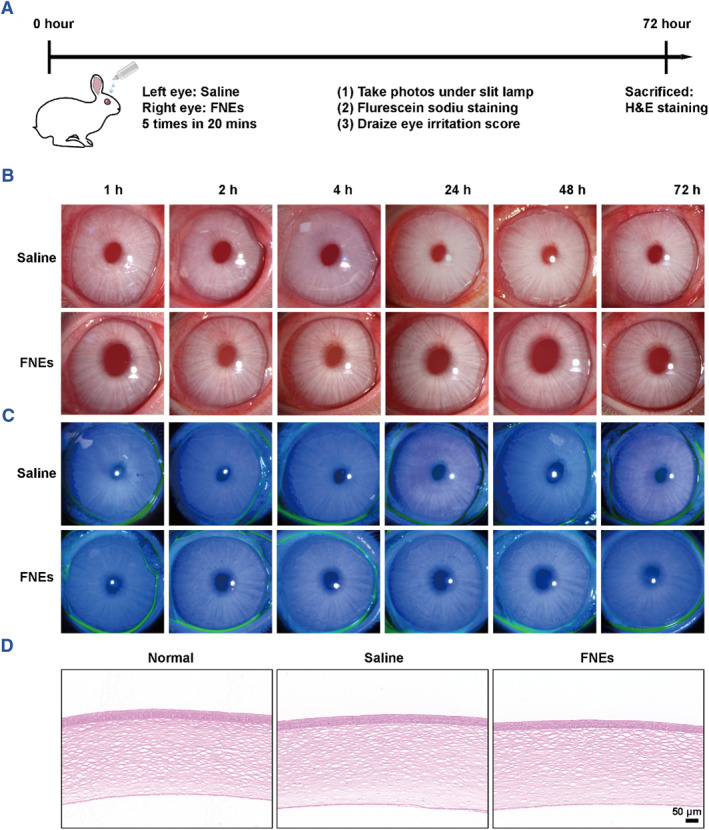
In vivo ocular irritation evaluation. (A) Schematic diagram of the details of rabbit irritation test in vivo. Representative photos (B) and fluorescein stained photos under slit‐lamp micrographs (C) of rabbit corneas with different treatments. (D) Representative H&E staining images of rabbit corneas after different treatments for 72 h.

Safety has always been a primary concern in laboratory and clinical studies of ocular formulations. To further evaluate the ocular irritancy of FNEs, the rabbit eye irritation test was performed using slit‐lamp microscopy and scored in reference to the Draize test.^52^ (Figure 6A). Rabbits were chosen for the study because their eyes are more sensitive to stimuli than human eyes. As shown in Figure 6B–D, the rabbits' eyes were intact with normal morphology and corneal structures, and there were no obvious epithelial defects and inflammatory cell infiltration after the instillation of the saline and FNEs solutions. The Draize test scores of the FNEs group are nearly the same as those of the saline group (Table S1). This result verifies that FNEs are not irritating to the eyes, indicating that FNEs hold great potential for clinical application.

## 4 CONCLUSION

In summary, we achieved FNEs with antibacterial and anti‐inflammatory ability for effective treatment of bacterial keratitis. FNEs could mimic POD activity and spontaneously catalyze H_2_O_2_ to produce much more ROS at the infection site to eliminate bacteria without generating drug resistance. Meanwhile, the FNEs exhibited distinguished biocompatibility to ocular cells and tissue. Apparently, the results have demonstrated that FNEs exhibited superior therapeutic potential in the mice bacterial keratitis model in comparison to conventional antibiotics. Our work provides insight into the great scientific value of the FNEs and imparts them with promising clinical applications.

## AUTHOR CONTRIBUTIONS

Xiwen Geng: Data curation; funding acquisition; methodology; visualization; validation; writing – original draft. Nan Zhang: Methodology. Zhanrong Li: Conceptualization; funding acquisition. Mengyang Zhao: Funding acquisition; visualization; Writing – review & editing. Hongbo Zhang: Supervision; writing – review & editing. Jingguo Li: Conceptualization; funding acquisition; writing ‐ review & editing.

## CONFLICT OF INTEREST STATEMENT

Hongbo Zhang is an executive editor for *Smart Medicine* and was not involved in the editorial review or the decision to publish this article. All authors declare that there are no competing interests.

## ETHICS STATEMENT

All protocols for animal assays were approved by the Committee on the Ethics of Animal Experiments of Henan Eye Hospital. All animals in our research were treated in strict accordance with the Association for The Study of Vision and Ophthalmology's Declaration on the Use of Animals in Ophthalmology and Vision Research.

## Supporting information

Supporting Information S1
