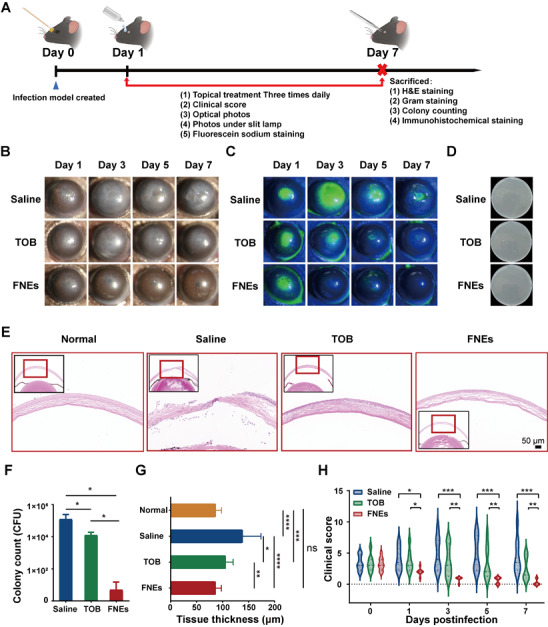# Correction to “Iron‐Doped Nanozymes With Spontaneous Peroxidase‐Mimic Activity as a Promising Antibacterial Therapy for Bacterial Keratitis”

**DOI:** 10.1002/smmd.70030

**Published:** 2026-01-09

X. Geng, N. Zhang, Z. Li, M. Zhao, H. Zhang, J. Li, *Smart Med*. **2024**, *3*(2), e20240004.

In Figure 4B, the cornea photo of the saline group on Day 5 was erroneously placed in the panel labeled Day 7, and the original photo intended for Day 7 was omitted. We have now corrected the figure by replacing the mislabeled photo with the corresponding Day 7 cornea photo. This error did not affect the statistical conclusions or overall interpretation of the study.

We sincerely apologize for this error.