# Supplementary material for: Iron‐doped nanozymes with spontaneous peroxidase‐mimic activity as a promising antibacterial therapy for bacterial keratitis
Source: Smart Med. 2024 May 22;3(2):e20240004. doi: 10.1002/SMMD.20240004 (PMC11236036; doi:10.1002/SMMD.20240004)
Supplement: Supplementary file 1 — Supporting Information S1 [file SMMD-3-e20240004-s001.docx]

**Title**: Iron-doped nanozymes with spontaneous peroxidase-mimic activity as a promising antibacterial therapy for bacterial keratitis

**Authors**: Xiwen Geng, Nan Zhang, Zhanrong Li, Mengyang Zhao, Hongbo Zhang, Jingguo Li

***Supplementary methods:***

**1. Biocompatibility studies**

The cytotoxicity tests of FNEs were evaluated in human corneal epithelial cells and human conjunctival epithelial cells using CCK-8 assay. The cells were seeded onto 96-well plates at a density of 6000 cells per well and cultured in 5% CO_2_ at 37 ^◦^C for 24 h. FNEs was diluted with the growth medium (DMEM/F12) to give final concentrations of 0-45 μg/mL. The media were replaced with 100 μL of the pre-prepared samples. After 24 h of incubation, the medium was removed and a fresh medium containing 10% CCK-8 solution was added to each well for an additional 1 h incubation. Then, the optical density (OD values) at 450 nm wavelength was recorded using the Cytation5 Microplate Reader (Biotek Winooski, Vermont, USA).

**2. Determination of growth kinetics and growth inhibition curves.**

Growth inhibition curve analyses were performed by culturing *S. aureus* in TSB. Plate-grown bacteria were inoculated into TSB medium with a preliminary OD_600_ of 0.1, and cultured for another 8 h at 37℃ with shaking. FNEs (tobramycin) was added to the broth to a final concentration of 31.25 (150) μg/mL. The CFU were counted at different time intervals (0, 0.5, 1, 2, 4, 6, and 8 h). The growth kinetics of strains treated with FNEs and TOB were compared with that of the control group. The growth profiles were monitored in TSB medium with a preliminary OD600 of 0.1 and then cultured for another 72 h at 37℃ with shaking. The records were taken every 12 h by determining the OD600 of the test strains. The values stated are the mode values from at least three biological replicates performed in at least three independent occasions.

**3. Morphological analyses of bacteria**

For bacteria observation, scanning electron microscopy (SEM) was used. The bacteria cells treated with or without 31.5 μg/mL FNEs (10 μM, H_2_O_2_) were fixed on glass slides with 4% paraformaldehyde for 15 min at room temperature, rinsed with PBS, then dried overnight. The samples were gold sputter-coated and imaged using a scanning electron microscope (Zeiss Sigma 500, Germany).

**4. Membrane destruction assays.**

The membrane destruction is determined by measuring the concentration of DNA and protein leaked from fungal cells in the medium. The concentration of the DNA was determined using a spectrophotometer (NanoDrop 2000, USA), and the protein concentration was determined using BCA Protein Assay kit (Beijing Solarbio Science & Technology Co., Ltd. China).

**5. *In vivo* irritation test**

The rabbit eye irritation test was performed with reference to Draize test. Three healthy New Zealand white rabbits (2.5–3.5 kg, 3 months) were used in the test. The right eyes were given 20 μL FNEs (50 μg/mL) once at 5-minute intervals, and five times within 20 minutes, and the left eyes were given saline as control. At 0, 1, 2, 4, 24, 48, and 72 h after administration, each eye was photographed using slit-lamp microscopy, and rated for irritation according to the Draize rule. After 72 h, the rabbits were sacrificed and the eyeball tissues were collected for H&E staining.

***Supplementary figures:***


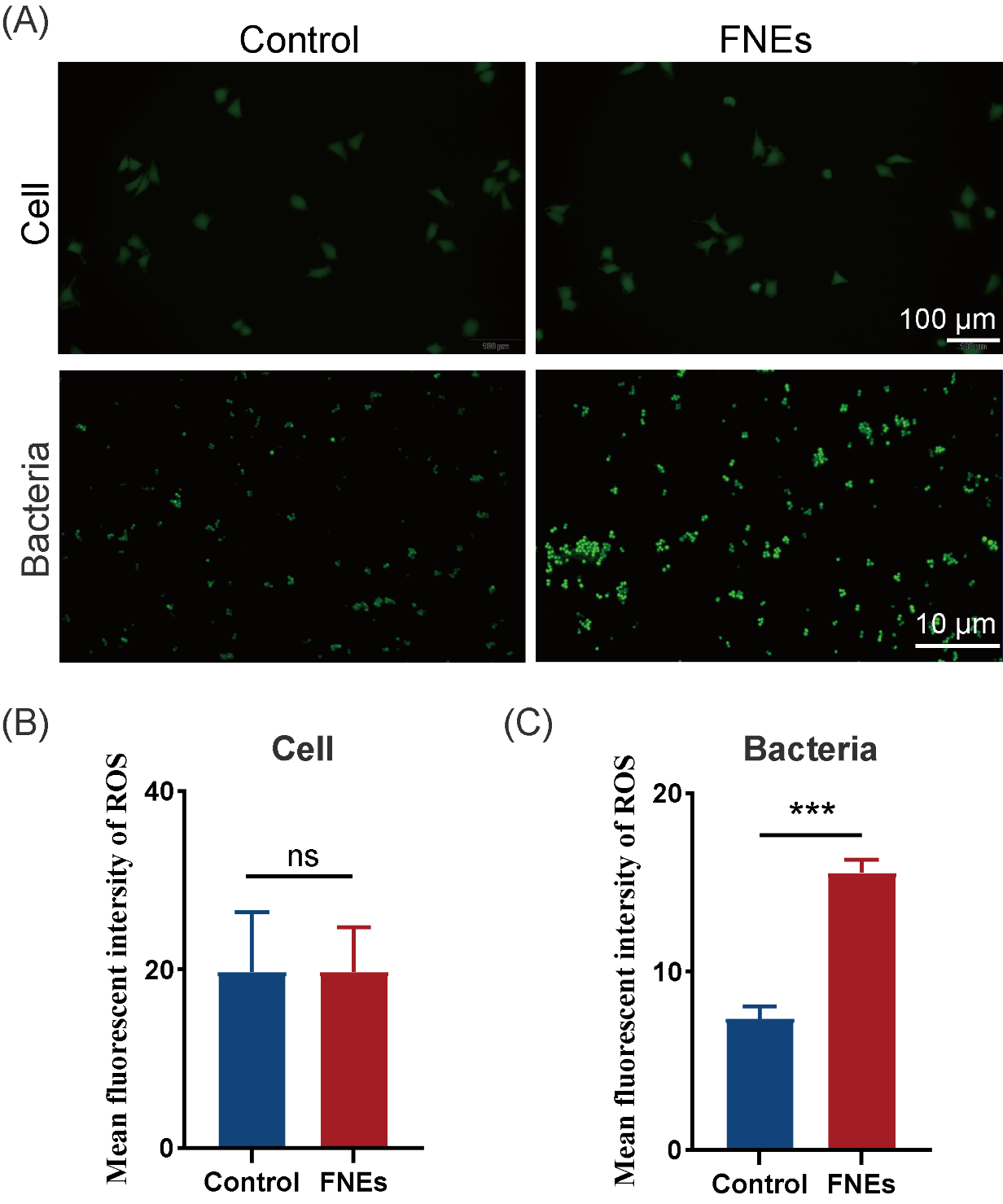


**Figure S1**. (A) Fluorescent images of ROS in the corneal epithelial cells and bacteria treated with or without FNEs (31.25 μg/mL). ROS fluorescence intensity of cell (B) and bacteria (C). Asterisks indicate significant differences (t-test, ***p < 0.001).


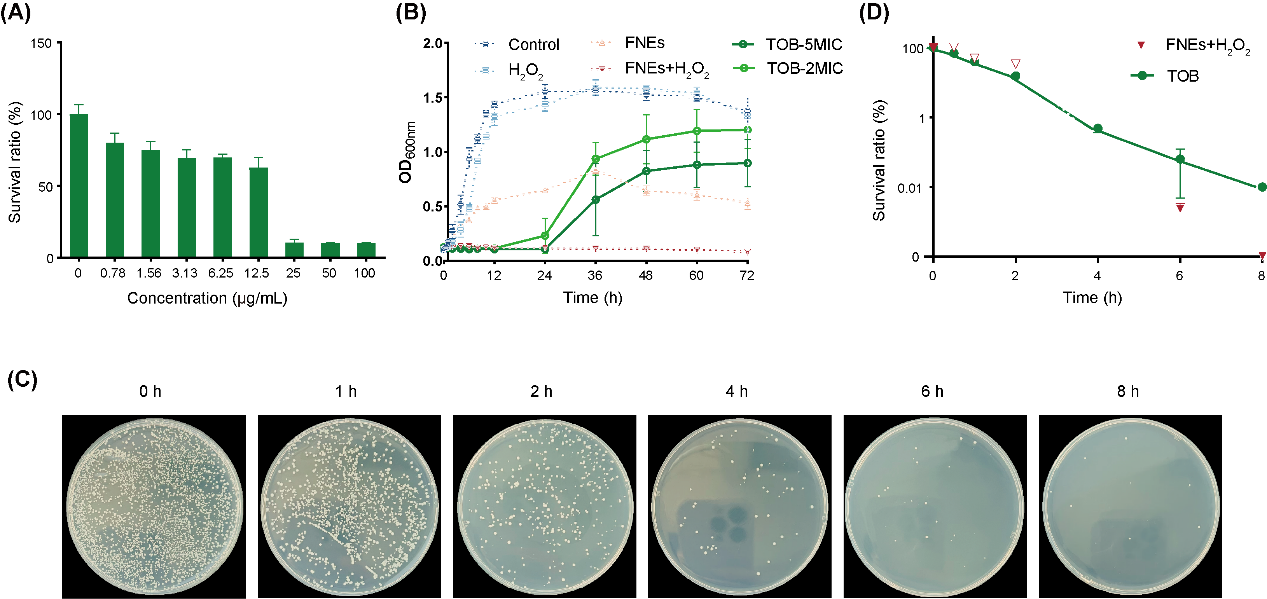


**Figure S2**. Antibacterial effect of TOB *in vitro*. (A) MICs of TOB. (B) Growth kinetics of *S. aureus* with different treatments, (C) the corresponding growth-inhibit curve, and (D) the corresponding plate counting assays.


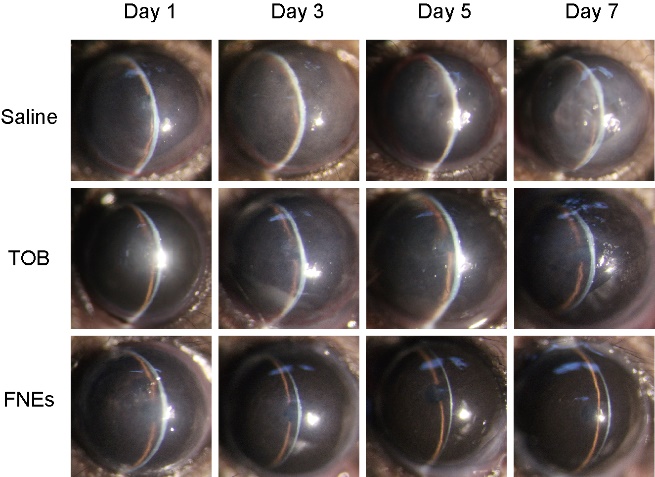


**Figure S3**. Representative photos of mice cornea under slit-lamp micrographs.


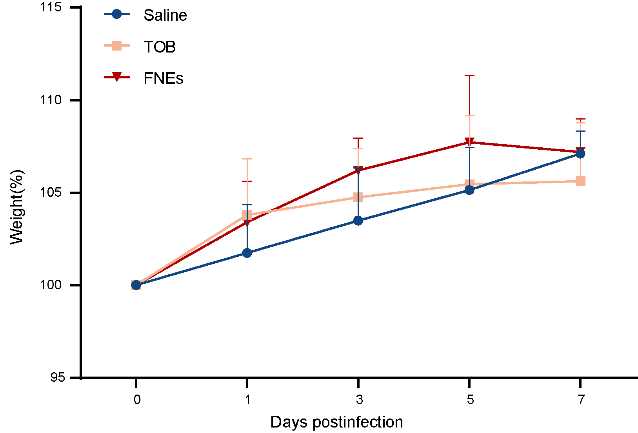


**Figure S4**. Body weight changes of mice during 7 days of different treatments.

**Table S1**. Scores of ocular irritation evaluation.

|  | Irritation test score | | | | | |
| --- | --- | --- | --- | --- | --- | --- |
|  | 1 | | 2 | | 3 | |
|  | L | R | L | R | L | R |
| 1 h | 0 | 0 | 1 | 1 | 1 | 0 |
| 2 h | 0 | 0 | 1 | 0 | 1 | 0 |
| 4 h | 0 | 0 | 1 | 1 | 1 | 1 |
| 24 h | 0 | 0 | 0 | 0 | 0 | 0 |
| 48 h | 0 | 0 | 0 | 0 | 0 | 0 |
| 72 h | 0 | 0 | 0 | 0 | 0 | 0 |

*0-3 no irritation, 4-8 mild irritation, 9-12 moderate irritation, 13-16 severe irritation
